# The Clinical Value of Capillary Blood Cartridge-Based Testing in Neonatal Jitteriness: A Re-Evaluation of the Diagnostic Approach

**DOI:** 10.3390/children12040510

**Published:** 2025-04-16

**Authors:** Assaf Regev, Rasha Srour, Laurence Mangel, Dror Mandel, Jacky Herzlich, Anat Lavie, Ronella Marom

**Affiliations:** 1 Pediatric Infectious Diseases Unit, Dana Dwek Children’s Hospital, Tel Aviv Medical Center, Tel Aviv 6423906, Israel; asafr@tlvmc.gov.il; 2 Faculty of Medical & Health Sciences, Tel Aviv University, Tel Aviv 6997801, Israel; rasha.srour@icloud.com (R.S.); drorm@tlvmc.gov.il (D.M.); jackyh@tlvmc.gov.il (J.H.); anatr@tlvmc.gov.il (A.L.); 3 Department of Neonatology, Dana Dwek Children’s Hospital, Tel Aviv Medical Center, Tel Aviv 6423906, Israel; laurencem@tlvmc.gov.il; 4 Department of Obstetrics and Gynecology, Lis Hospital, Tel Aviv Medical Center, Tel Aviv 6423906, Israel

**Keywords:** jitteriness, neonate, hypoglycemia, selective serotonin reuptake inhibitors, emergency cesarean sections

## Abstract

Objective: This study assessed the utility of capillary blood cartridge-based analysis in evaluating neonatal jitteriness (NJ). Methods: In this retrospective study, we compared outcomes between neonates (37–41 weeks of gestation) diagnosed with neonatal jitteriness (NJ) within the first 72 h of life and a control group of healthy neonates (GA 37–41 weeks) with an uneventful perinatal course and no signs of jitteriness. Results: Each group included 101 neonates. Jittery neonates had a higher proportion of males (70.3% vs. 50.5%, *p* = 0.004), a lower mean gestational age (38.8 vs. 39.2 weeks, *p* = 0.002), and a higher rate of emergency cesarean deliveries (14.9% vs. 3.0%, *p* = 0.003). The logistic regression identified male sex (OR = 2.5, *p* = 0.007) and in utero selective serotonin reuptake inhibitor (SSRI) exposure (OR = 9.0, *p* = 0.005) as significant risk factors for NJ. The capillary blood parameters, except glucose levels, did not differ significantly between the neonates admitted to the NICU and those discharged. Hypoglycemic jittery neonates were 10 times more likely to require NICU admission compared to their non-hypoglycemic counterparts (OR = 10.9, 95% CI: 2–59.5, *p* = 0.006). Conclusions: Point-of-care glucose testing using a bedside glucometer may be sufficient for the evaluation of neonatal jitteriness, as capillary blood cartridge-based testing did not offer an additional diagnostic value. What is Known: NJ is often viewed as a self-resolving benign phenomenon; however, in certain cases, it can be an indicator of an underlying pathology. There is substantial evidence linking the maternal use of SSRIs or SNRIs during pregnancy with the occurrence of NJ in newborns as well as an association between hypoglycemia and NJ. What is New: This study is the first to evaluate the clinical utility of systematic capillary blood cartridge-based testing in jittery neonates using a relatively large cohort. Male neonates were disproportionately represented among cases of NJ. Healthy neonates with jitteriness had normal electrolytes, with hypoglycemia as the only concern. A glucometer test may suffice for evaluation, but those who are small for their gestational age or have initial hypoglycemia require a routine follow-up due to a higher risk of NICU admission.

## 1. Introduction

Neonatal jitteriness (NJ) is a clinical motor phenomenon described as a generalized, symmetrical coarse tremor of high frequency and a low amplitude [1]. It is characterized by a tremor that ceases upon passive flexion and is the most common involuntary movement of healthy full-term neonates [2]. The prevalence is estimated at up to 44% of newborns in their first days of life [3].

Despite being so prevalent, there is a paucity of publications on the topic in the past 20 years, and most known data originate from retrospective cohorts from the late 1980s to the early 2000s [3,4,5]. Many questions are left unanswered regarding NJ, some of which carry significant implications for the diagnosis and evaluation of jittery neonates.

NJ can have varying implications; while it is often viewed as a self-resolving benign phenomenon, in some cases it may also be considered as a sign of a potential pathology [3,6,7]. Such possible associations include hypoxic–ischemic encephalopathy, intracranial hemorrhage, sepsis, an electrolyte imbalance (mostly hypocalcemia or hypomagnesemia), hypoglycemia, withdrawal syndrome as well as maternal hyperthyroidism and congenital heart defects, polycythemia, and even hypovitaminosis D [3,8,9,10,11,12]. Regardless of its cause, jitteriness in healthy infants significantly decreases by the second week of life and typically disappears after that period [3].

To date, no recommendations have been made for assessing NJ. The decision to evaluate for NJ remains subjective and depends on the clinical judgment of pediatricians and neonatologists.

At our institute, the standard approach for evaluating all cases of NJ involves capillary blood cartridge-based testing to assess for hypoglycemia, hypocalcemia, and other electrolyte abnormalities. Markers of perinatal stressor events, such as asphyxia or sepsis, are also evaluated using lactate and base excess measurements.

The objective of this study was to evaluate the diagnostic relevance and clinical necessity of this testing approach in otherwise healthy neonates presenting with NJ. By assessing the utility of these tests, we aimed to support a more targeted diagnostic strategy and minimize unnecessary investigations.

## 2. Materials and Methods

### 2.1. Participants and Study Design

In this retrospective study, we reviewed medical records of neonates admitted to the nursery department between December 2020 and December 2021. The local institutional review board approved this study (1096-20-TLV) and waived the need for informed consent due to its retrospective character. This study was carried out in accordance with Good Clinical Practice guidelines and the Declaration of Helsinki. Among all neonates who underwent capillary blood cartridge-based testing, those with a gestational age (GA) from 37 to 41 weeks, diagnosed with jitteriness within the first 72 h of life but otherwise healthy, were identified. Diagnosis of jitteriness was made by the permanent medical staff which includes a pediatrician, neonatologist, and medical resident. For each included jittery neonate, the next consecutive healthy neonate (GA 37–41 weeks) with an uneventful perinatal course and no signs of jitteriness was selected as a control after reviewing medical records.

### 2.2. Data Collection

Demographic, clinical, and laboratory data of the neonates included mode of delivery, GA, birth weight (BW), gender, weight for gestational age (appropriate for gestational age (AGA), small for gestational age (SGA), large for gestational age (LGA), Apgar scores, head circumference, Neonatal Intensive Care Unit (NICU) admission, body temperature, blood pH, base excess, lactate, glucose, sodium, potassium, and calcium. Capillary blood tests (heel puncture) were performed in a cartridge-based blood gas system. Maternal data included age; drug use during pregnancy, including selective serotonin reuptake inhibitor (SSRI) or serotonin and norepinephrine reuptake inhibitor (SNRI); and gestational diabetes mellitus.

### 2.3. Statistical Analysis

Continuous data were expressed as means ± standard deviations (SD) or median and interquartile (Q1–Q3). Categorical parameters were expressed as frequency and percentages. Normality was assessed by Shapiro–Wilk tests. Chi-squared tests or Fisher’s exact tests were used for categorical variables, and Mann–Whitney U test, Kruskal–Wallis test (adjusted for multiple comparisons using the Bonferroni correction), or Student’s *t*-test were used for continuous variables to compare outcomes between groups, as appropriate. Adjusted odds ratios (ORs) were calculated with 95% confidence intervals (CIs) to assess the association between maternal and/or neonatal variables and the likelihood of presenting with NJ and NICU admission for jittery neonates. The IBM SPSS software for windows, version 29, was used for all statistical analyses. Statistical tests were two-sided and *p* < 0.05 was considered statistically significant.

## 3. Results

**Figure 1:**
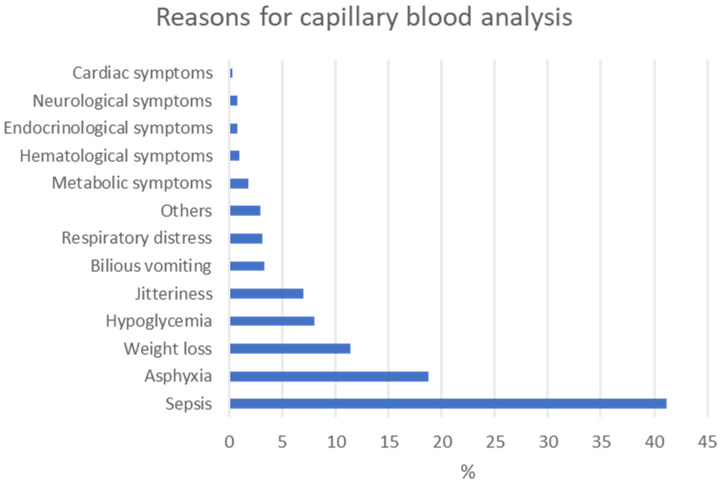
Reasons for capillary blood analysis.

Out of 1497 term neonates who underwent capillary blood cartridge-based testing, 105 (7%) exhibited jitteriness as the only symptom, making it the fifth most common reason for performing this test (Figure 1).

**Figure 2:**
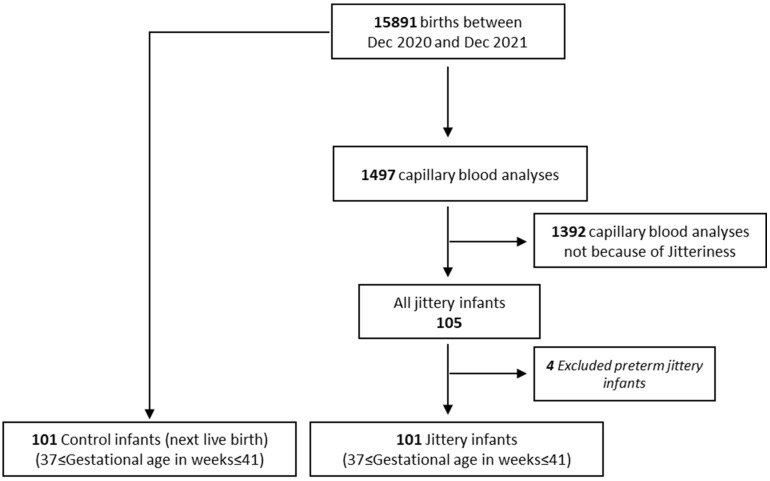
Flowchart of cohort.

Of these, all jittery neonates who met the inclusion criteria (N = 101) were included in the study cohort. Both the study and control groups are shown in Figure 2.

The demographic and clinical data of the study cohort are summarized in Table 1. The groups did not differ in terms of the maternal age, gestational diabetes mellitus, BW, head circumference, weight for gestational age, and Apgar scores. However, jittery neonates had a lower median GA compared to those in the control group (38.8 vs. 39.2 weeks, *p* = 0.002). Additionally, the rate of emergency cesarean sections was five times higher for jittery neonates than for non-jittery neonates (14.9% vs. 3%, Adjusted *p* = 0.003). A total of 21.8% of mothers in the jittery group reported drug use during pregnancy. The majority of jittery neonates were males, in contrast to the control group (70.3% versus 50.5%, *p* = 0.004), and 17.8% of the neonates in the jittery group were exposed to SSRIs/SNRIs during pregnancy, as opposed to only 2% in the control group (*p* < 0.001).

Moreover, the logistic regression analysis controlling for potential confounders, such as the mode of delivery, GA, BW, gender, and maternal use of SSRIs during pregnancy, showed that male neonates had 2.5 times higher odds of presenting with NJ compared to females (OR = 2.5, 95% CI: 1.3–4.8, *p* = 0.007). Additionally, neonates exposed to SSRIs/SNRIs in utero were significantly more likely to present with NJ (OR = 9, 95% CI: 1.9–42.1, *p* = 0.005). The mode of delivery did not show any impact on the likelihood of NJ. The overall model was statistically significant (χ^2^(6) = 35.3, *p* < 0.001).

Approximately 10% of the jittery neonates were admitted to the NICU and were compared to those discharged home (Table 2). The capillary blood cartridge-based testing of all jittery neonates showed that their blood parameters, except for glucose levels, were within normal ranges. There were no significant differences between the groups in terms of the mode of delivery, exposure to SSRI/SNRI, gestational diabetes mellitus, gender, GA, BW, head circumference, body temperature, blood pH, sodium, potassium, calcium, and base excess measurements. While the median blood lactate concentration was significantly higher in neonates admitted to the NICU compared to those discharged home (3.6 vs. 3 mmol/L, *p* = 0.043), the values remained within the normal range. Notably, although not statistically significant when adjusted for multiple analyses, 30% of the jittery neonates admitted to the NICU were SGA, compared to only 5.5% of those discharged home. They also had a significantly higher rate of hypoglycemia (50% vs. 12.1%, *p* = 0.008). In a logistic regression model assessing the odds of a jittery neonate being admitted to the NICU, using the gender, GA, lactate level, weight for gestational age, and hypoglycemia as predictors, jittery neonates with hypoglycemia were found to be 10 times more likely to be admitted to the NICU compared to non-hypoglycemic neonates (OR = 10.9, 95% CI: 2–59.5, *p* = 0.006). Additionally, for each one-unit increase in lactate levels, the likelihood of NICU admission doubled (OR = 2.1, 95% CI: 1.1–3.8, *p* = 0.019). The overall model was statistically significant (χ^2^(6) = 19.7, *p* = 0.003), explaining 37.8% of the variance in NICU admissions (R^2^ = 0.378) and correctly classifying 91.8% of cases.

## 4. Discussion

This study is the first to evaluate the usefulness of systematic capillary blood cartridge-based testing in jittery neonates, using a relatively large cohort. Our findings indicated that neonates presenting with jitteriness but who were otherwise healthy had electrolyte levels within the normal range. However, those with hypoglycemia were more likely to be admitted to the NICU, and this risk increased with each unit rise in lactate levels. Most of these neonates had lactate levels ≤5.1 mmol/L within the first 72 h of life and a normal 5 min Apgar score, suggesting that the elevated risk of NICU admission associated with these lactate levels is clinically insignificant. Therefore, NJ may raise suspicion of being pathological when it is accompanied by hypoglycemia, which remains a strong predictor for NICU admission [13,14,15]. Additionally, our findings revealed that jitteriness was more common in male neonates. While a definitive pathophysiological explanation remains unclear, some studies have suggested gender differences in response to perinatal insults, hormonal fluctuations, and neurotransmitter distribution as potential factors [16,17]. This is the first data on the male predominance regarding jitteriness in neonates.

We found that in an utero exposure to SSRIs/SNRIs increases the risk of NJ. The link between maternal SSRI/SNRI use during pregnancy and NJ has been widely documented [18,19,20,21]. Given the steadily increasing prevalence of SSRI/SNRI use among pregnant women, it has probably become a significant contributor to NJ [22]. Additionally, the proportion of SGA jittery neonates admitted to the NICU was higher compared to those discharged home, consistent with findings from previous studies [23].

Capillary blood cartridge-based testing is a fast and relatively comprehensive method to evaluate symptomatic neonates. However, it has limitations regarding accuracy, it requires specific skills [24,25,26], is more time-consuming and expensive, and, most importantly, is painful for the neonate and alarming for the parents. Given the number of tests at our institute solely for NJ, which ranked as the fifth most common reason for laboratory assessment in neonates after sepsis, asphyxia, weight loss, and hypoglycemia, and considering that capillary blood cartridge-based testing in newborns with a benign tremor has shown limited diagnostic value, our approach to evaluating the clinical implications of NJ is currently under revision.

It is suggested that this critical transition from fetal to neonatal life may cause a period of physiological instability, during which the brain must adjust to the new environment. This adjustment period, which lasts several days, may result in temporary abnormalities in neonatal movement, as seen in jitteriness [27]. A recent study by Xu et al. supports this view, showing that the earliest appropriate time for a neurobehavioral assessment in newborns is at least 20 h after birth [28]. Our overarching goal remains the reduction in unnecessary testing, particularly when blood glucose levels can be reliably assessed using a bedside glucometer. Point-of-care (POC) glucometer-based glucose monitoring is a widely accepted and routinely employed method for evaluating blood glucose in the neonatal population. Although both glucometer readings and cartridge-based glucose measurements rely on capillary blood samples, differences in analytical techniques and calibration can result in a variability between the two approaches. Moreover, the accuracy of POC glucometers can vary across different devices, highlighting the importance of selecting reliable and validated tools for neonatal glucose monitoring [29,30].

Our study is the first in decades to explore neonatal jitteriness. However, its retrospective design presents limitations, including a reliance on the accuracy and completeness of existing medical records and the potential for selection bias. Additionally, variability in the diagnosis of neonatal jitteriness may have occurred due to differences in the composition of the permanent medical staff that were present on any given day.

## 5. Conclusions

Neonatal jitteriness is a common motor phenomenon with a wide range of presentations, from a benign physical examination finding to an indicator of severe neurological or endocrinological emergencies. We suggest that an initial glucose test using a glucometer may be sufficient for evaluating otherwise healthy neonates presenting with jitteriness.

## Figures and Tables

**Table 1:** The maternal and neonatal characteristics of the study cohort.

Characteristic	Jittery Group N = 101	Control Group N = 101	*p* Value
Maternal age, y	33.7 ± 5 (22.7–46.8)	33.2 ± 5.1 (21.5–46.8)	0.424
SSRI/SNRI use during pregnancy	18 (17.8)	2 (2)	<0.001
Gestational DM	16 (15.8)	10 (9.9)	0.176
Mode of delivery			0.009
Spontaneous delivery	68 (67.3)	82 (81.2)	
Elective cesarean section	18 (17.8)	16 (15.8)	
Emergency cesarean section	15 (14.9)	3 (3)	
Gender—Male	71 (70.3)	51 (50.5)	0.004
GA, w	38.8 ± 0.9 (37–41)	39.2 ± 1.2 (37–41)	0.002
Birth weight, g	3206.2 ± 496.4 (2020–4468)	3307.1 ± 473.7 (2304–4415)	0.141
Head circumference, cm	34.1 ± 1.4 (31–37.5)	34 ± 1.4 (30.5–37)	0.412
AGA	82 (81.2)	80 (79.2)	0.798
SGA	8 (7.9)	7 (6.9)	
LGA	11 (10.9)	14 (13.9)	
Apgar score at 1 min	9 [9, 9]	9 [9, 9]	0.161
Apgar score at 5 min	10 [10, 10]	10 [10, 10]	0.656
Neonatal Intensive Care Unit admission	10 (9.9)	0	NA

Data are expressed as mean ± standard deviation (range), median [Q1, Q3], or n (%). SSRI: selective serotonin reuptake inhibitor; SNRI: serotonin norepinephrine reuptake inhibitor; DM: diabetes mellitus; GA: gestational age; AGA: appropriate for gestational age; SGA: small for gestational age; LGA: large for gestational age; NA: not assessed.

**Table 2:** Comparison between jittery neonates admitted to NICU with those discharged home.

Characteristic	Admitted to NICU N = 10	Discharged Home N = 91	*p* Value
Maternal age, y	34.2 ± 6.8 (23.2–46.8)	33.7 ± 4.8 (22.7–46.8)	0.759
SSRI/SNRI use during pregnancy	0	18 (19.8)	0.202
Gestational DM	1 (10)	15 (16.5)	>0.99
Mode of delivery			0.465
Spontaneous delivery	6 (60)	62 (68.1)	
Elective cesarean section	1(10)	17 (18.7)	
Emergency cesarean section	3 (30)	12 (13.2)	
Apgar scores			
1 min	9 [9, 9]	9 [9, 9]	0.853
5 min	10 [9.8, 10]	10 [10, 10]	0.417
Gender—Male	9 (90)	62 (68.1)	0.274
GA, w	38.5 [37, 39.3]	39 [38, 39]	0.427
Birth weight, g	3134.5 ± 696.5 (2020–4280)	3214.1 ± 474 (2100–4468)	0.633
AGA	6 (60)	76 (83.5)	0.049
SGA	3 (30)	5 (5.5)	
LGA	1 (10)	10 (11)	
Head circumference, cm	34 ± 2 (30.5–36.8)	33.9 ± 1.3 (30.5–37)	0.100
pH	7.4 [7.3, 7.4]	7.3 [7.3, 7.4]	0.534
Base excess, mmol/L	−3.5 [−5.5, −2]	−3 [−4, −1]	0.241
Lactate, mmol/L	3.6 [3, 5.1]	3 [2.3, 3.5]	0.043
Blood glucose, mg/dL	44.5 [37.3, 50.5]	59 [47, 68]	0.012
Hypoglycemia (According to age in hours)	5 (50)	11 (12.1)	0.008
Blood sodium, mmol/L	137.4 ± 2.6 (133.5–143.3)	138.8 ± 3.1 (131.3–147)	0.176
Blood potassium, mmol/L	5.4 [4.9, 5.5]	5.2 [4.8, 5.7]	0.986
Blood ionized calcium, mmol/L	1.2 [1.2, 1.3]	1.2 [1.2, 1.3]	0.274
Body temperature, °C	36.9 ± 0.2 (36.6–37.3)	36.9 ± 0.4 (36–37.8)	0.841

Data are expressed as mean ± standard deviation (range), median [Q1, Q3], or n (%). SSRI: selective serotonin reuptake inhibitor; SNRI: serotonin norepinephrine reuptake inhibitor; DM: diabetes mellitus; GA: gestational age; AGA: appropriate for gestational age; SGA: small for gestational age; LGA: large for gestational age.

## Data Availability

The original contributions presented in this study are included in the article. Further inquiries can be directed to the corresponding author(s).
